# Interventions and Implementation Strategies for Preventing Occupational Contact Dermatitis: A Scoping Review

**DOI:** 10.1111/cod.70113

**Published:** 2026-02-15

**Authors:** Jonathan A. G. Jonker, Sietske J. Tamminga, Felicia S. Los, Parel M. V. Janse, Sanja Kezic, Henk F. van der Molen, Julitta S. Boschman

**Affiliations:** ^1^ Amsterdam UMC location University of Amsterdam Public and Occupational Health Amsterdam the Netherlands; ^2^ Amsterdam Public Health Research Institute, Societal Participation and Health Amsterdam the Netherlands

**Keywords:** eczema, health surveillance, occupational disease, prevention, protection, skin disease

## Abstract

Numerous preventive measures for occupational contact dermatitis (OCD) have been evaluated, but their effectiveness varies, suggesting that contextual factors and corresponding implementation strategies are important. This scoping review aimed to identify preventive interventions for OCD and explore their implementation strategies and outcomes. We searched five databases (January 2000–May 2024) for studies on preventive interventions, scoping intervention content and implementation strategies. In total, 111 articles describing 79 interventions were included, which involved components of education, personal protective equipment, skin care, workplace adaptations and combinations. Most studies were conducted among healthcare workers, hairdressers or in mixed occupations. Implementation strategies targeted individual workers with educational sessions, individual advice or consults, organisations with participatory working groups, role models or communication tools, and facilitated clinical dermatological care. Implementation outcomes were reported for 11 programs. All reported appropriateness and 10 reported acceptability to be positive. Adoption, feasibility, fidelity (adherence), costs, penetration (reach) and sustainability of preventive intervention implementation were assessed in a limited number of programs. Overall, limited evidence precluded firm conclusions on implementation outcomes. To strengthen prevention, systematic evaluation of implementation outcomes is needed.

## 1 Introduction

Occupational contact dermatitis (OCD) is a frequently reported occupational disease [1]. OCD, typically affecting the hands, is particularly prevalent in occupations with frequent exposure to allergens and irritants. Estimated 1‐year prevalence reaches 27% among healthcare workers and 20% among hairdressers [2, 3]. However, the true prevalence is likely underestimated due to underreporting. With its chronic course and considerable impact on sickness absence, healthcare costs and job loss or change, OCD requires prevention‐focused approaches, as treatment alone cannot sufficiently reduce its burden at either the individual or organisational level.

Prevention of occupational diseases in high‐risk occupations can be approached at different levels, as illustrated by the Three‐Level Prevention Model [4, 5], distinguishing between primary prevention for workers at risk, secondary for those with early symptoms and tertiary for those unable to work due to disease. Various reviews investigated OCD prevention [5, 6, 7, 8, 9] and reported organisational and personal preventive measures. However, these preventive measures were implemented differently. For example, by distributing skin care products directly to workers or at the workplace and with or without accompanying guidance and education on their use [6, 7]. Other reviews focused on OCD prevention in specific occupational groups [8, 9] or within particular national contexts [5]. In general, preventive measures can be effective in OCD prevention to varying degrees, but currently an overview encompassing their implementation strategies does not yet exist.

Understanding implementation strategies is valuable to optimise the uptake of preventive strategies for OCD. Implementation research has identified multiple determinants influencing implementation with strategies operating at individual, organisational and system levels. Various implementation frameworks have been proposed, each emphasising different aspects of the implementation process [10]. Given this diversity, this review aims to map an overview of interventions and implementation strategies using the following implementation outcomes: *acceptability, adoption, appropriateness, feasibility, fidelity, implementation cost, penetration* and *sustainability* [11]. Information on effective implementation strategies is important to structure actions addressing work‐related risk factors through primary, secondary and tertiary prevention [12, 13, 14].

## 2 Methods

This scoping review was informed by the Joanna Briggs Institute's (JBI) Manual for Evidence Synthesis for Scoping Reviews [15]. The review protocol has been registered and published in the Open Science Framework (OSF) [16]. Deviations from the protocol included extracting and reporting the implementation strategy separately from the intervention measures and implementation outcomes.

### 2.1 Search Strategy

A medical information specialist searched five databases for relevant studies: MEDLINE, Embase, CDSR, CENTRAL and CINAHL. This search included standardised keywords (MeSH terms) as well as free text terms relating to dermatitis or skin diseases, occupation or work, and prevention or health program (see Supplementary File 1 for the full search strategy). Retrieved citations were imported into Covidence for screening [17]. The reference lists of relevant review articles were screened to identify additional publications.

### 2.2 Study Eligibility

Included articles targeted workers or apprentices and described interventions or preventive measures specific to OCD; either connected to the workplace or with a component occurring at the workplace. Only publications in English, Dutch, German or French from January 2000 to May 2024 were considered. Furthermore, articles solely promoting general or mental wellbeing, or targeting skin diseases other than OCD were excluded. Legislation and policy were disregarded.

**FIGURE 1:**
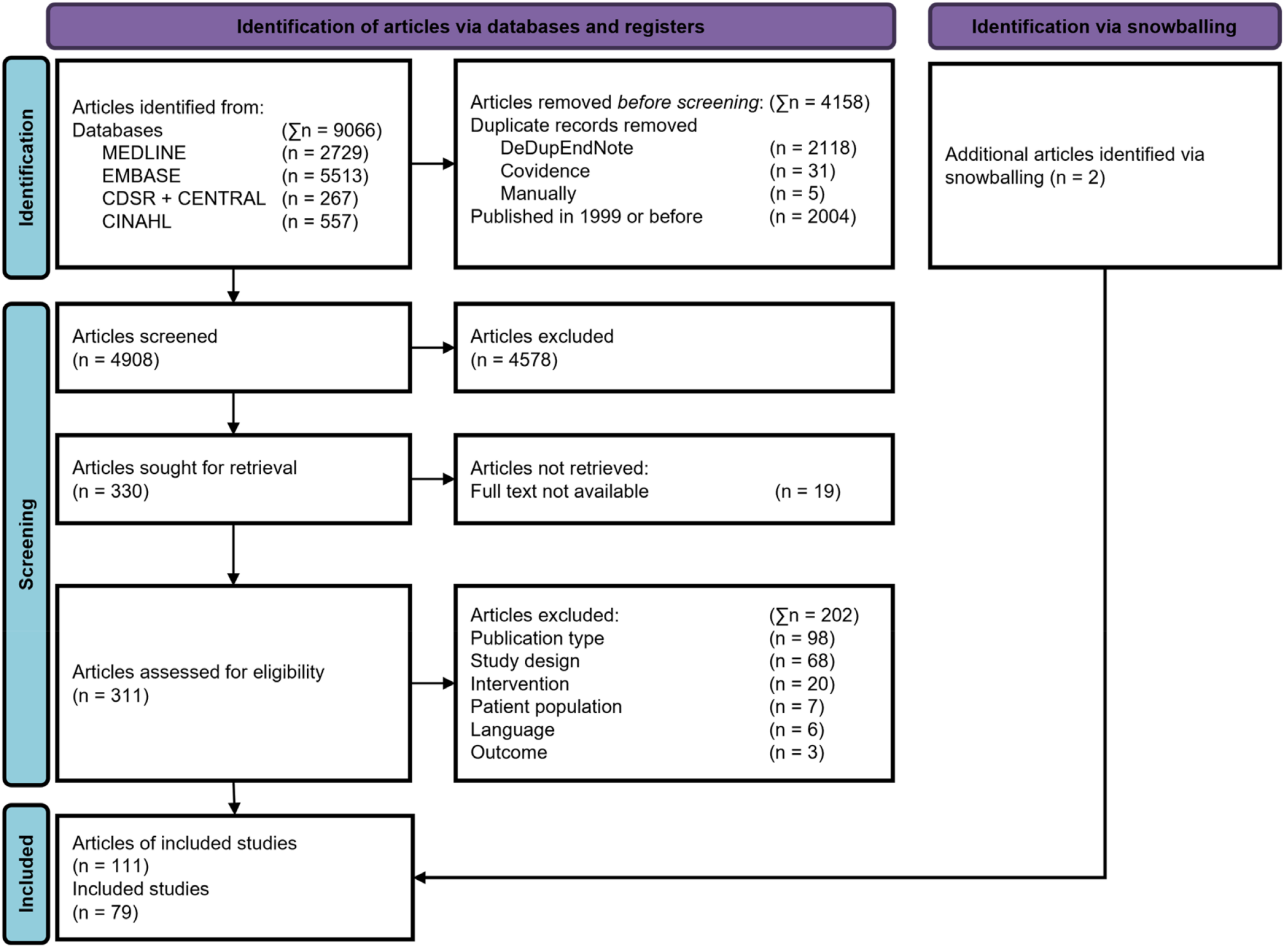
PRISMA flow diagram [18]. Studies were excluded for a number of reasons, with an unfitting publication type (e.g., congress abstract or supplementary file) or wrong study design (e.g., review article or prevalence study) being the most common. A number of included articles reported the same study and intervention, resulting in them being grouped during the data analysis.

Following a pilot test of 100 titles and abstracts, all articles were screened independently by two independent reviewers (JJ and one of JB, ST, FL, HM or SK). Potentially relevant articles were retrieved in full. Full text selection was done by two independent reviewers (JJ and one of JB, ST, FL, HM or SK). Reasons for exclusion were recorded and reported (Figure 1). Any disagreements that arose between the reviewers at each stage of the selection process were resolved through discussion or with an additional reviewer. The results of the search and the study inclusion process are presented in a PRISMA flow diagram (Figure 1) [18].

### 2.3 Data Extraction and Analyses

Following a pilot test of 10 articles, relevant study data was extracted by individual researchers (JJ, PJ, ST or JB) using a previously established extraction instrument (Supplementary File 3). In the case of uncertainty surrounding the extracted data, a second researcher was consulted for verification. For all included studies, their setting (country), target population, prevention type, preventive measures and outcomes were assessed and displayed in tabular format. Prevention was categorised as primary, secondary or tertiary. Primary prevention targeted individuals without symptoms or the entire workforce regardless of symptom status. Secondary prevention addressed active workers or sub‐populations already displaying symptoms of OCD, either within a single occupational group or across multiple groups. Tertiary prevention focused either on a sub‐population of a specific occupational group or mixed occupations that were unable to work due to OCD. Studies relevant to multiple categories were classified under all applicable categories.

For programs that aimed to research one or more of the implementation outcomes, the implementation strategies and information surrounding implementation outcomes were assessed. I available, a qualitative and quantitative representation was reported for each of the implementation outcomes: acceptability, adoption, appropriateness, feasibility, fidelity, implementation cost, penetration and sustainability [11]. The assessment was retroactively done by us based on the available published information. If no such information was available, as it was not assessed or reported by the original authors, this is reported.

## 3 Results

### 3.1 Results of the Search

The database search identified 9066 references, of which 4908 remained after removing duplicates. Following the screening of titles and abstracts, 311 full‐text articles were assessed for eligibility, resulting in the exclusion of 202 articles. Two articles were identified by snowballing. Ultimately, 111 articles describing 79 studies met the inclusion criteria (Figure 1). While all articles focused on interventions related to OCD, 11 distinct programs, containing 29 studies, reported on implementation outcomes.

### 3.2 Characteristics of the Included Studies

Of the included studies, 62 (78.5%) were conducted in Europe, primarily in Germany and Denmark, 11 (13.9%) in North America and 6 (7.6%) in the Middle‐East, Asia or Australia (Supplementary File 2). The studied populations included healthcare workers in 24 studies (30.4%), metal industry workers in 8 studies (10.1%), food handling and catering workers in 7 studies (8.9%) and hairdressers in 7 studies (8.9%). Other studied populations included cleaners and mechanics, as well as construction, production and print workers, all reported in two or fewer studies. Additionally, 25 studies (31.6%) included a mixed occupational group (Table 1). The majority of studies were cohort studies with 43 (54.4%) prospective and four (5.1%) retrospective cohorts. Sixteen studies (20.3%) were randomised controlled trials, seven (8.9%) had a pretest—posttest design, three (3.8%) were non‐randomised trials and six (7.6%) used other study designs (Table 1).

**TABLE 1:** Description of included interventions and outcomes.

References	Target population (Occupation)	Study sample^a^	Study design	Main prevention type	Intervention components					Outcomes			
					Education^b^	Skin care	PPE	Workplace adaptations	Other	Proximal (e.g., behaviour)	Trend^c^	Distal (e.g., symptoms, prevalence)	Trend^c^
Ahmed et al. [19]	Mechanics	106	Prospective cohort study	Primary	P,E		x			Knowledge, behaviour and PPE self‐efficiency	↑		
Bauer et al. [20, 21, 22]	Food or catering	55|91	Non‐randomised comparative trial	Primary					UVB hardening			Contact Dermatitis (CD) prevalence	↓
												Trans‐Epidermal Water Loss (TEWL)	—
	Food or catering	39|91		Primary	S,C,P	x	x			PPE and skin care usage	↑	CD prevalence	↓
												TEWL	↓
Baumeister et al. [23]	Metal workers	100	Prospective cohort study	Primary					Skin surveillance			Assessment skin condition	↑
Bregnhoj et al. [24, 25, 26]	Hairdressers	201|302	Prospective cohort study	Primary	S,P,E		x			Glove usage	↑	CD prevalence	↓
Brown et al. [27]	Print workers	22	Randomised controlled trial (RCT)	Primary					Skin checks	Skin care usage	↑	CD prevalence	↓
										PPE usage	↑	CD severity	↓
		30		Primary		x	x			PPE knowledge	↑		
		29		Primary	E					Behavioural change regarding exposure	—		
										Work practices	—		
		13		Primary				x					
Budd et al. [28]	Mixed	60	Implementation study	Primary	x								
Clemmensen et al. [29]	Cleaners	105	Prospective cohort study	Primary	C,P	x	x			Washing behaviour	↑	Skin symptoms	↑
										PPE usage	—		
										Skin care usage	—		
										Skin protection knowledge	↑		
Edelstam et al. [30]	Healthcare workers	4500	Pretest–posttest	Primary			x	x		Latex gloves usage	↓	CD prevalence	↓
Flyvholm et al. [31, 32, 33]^d^	Food or catering	205|439	Prospective cohort study	Primary	x			x		Glove usage	↑	CD prevalence	↓
										Skin care usage	↑		
Gallo et al. [34]	Healthcare workers	82	Pretest–posttest	Primary	x	x	x			Skin care usage	↑	Hand Eczema (HE) prevalence	—
										Risk awareness	↑		
										Extended glove usage	↓		
										Handwashing frequency	↓		
Geens et al. [35]	Hairdressers	11	Prospective cohort study (pilot)	Primary	P		x			Exposure during glove usage	↓		
Haughtigan et al. [36]	Hairdressers	170	Pretest–posttest	Primary	P,x					CD knowledge	↑		
										Glove usage	↑		
										Skin care usage	↑		
										Handwashing frequency	—		
Held et al. [37]	Healthcare workers (student)	61|46	RCT	Primary	C,P,E					Hand disinfectant usage	↑	CD prevalence	—
												TEWL	↓
										Handwashing frequency	—		
										Glove usage	—		
										Skin care usage	—		
Korniewicz et al. [38]	Healthcare workers	203|272	Prospective cohort study	Primary			x					Skin condition	—
												Skin irritation	↓
Lee et al. [39]	Food or catering	30	Prospective cohort study	Primary	P,E		x			Latex gloves knowledge	↑		
										Glove usage	↑		
Loffler et al. [40]	Healthcare workers	521	Prospective cohort study	Primary	C	x				Handwashing frequency	↓	Skin condition	↓
												HE prevalence	↓
Madan et al. [41, 42]^d^	Healthcare workers (student)	185|142	RCT	Primary	C,E,x	x				Handwashing frequency	↓	Skin condition	—
										Skin care usage	—	HE prevalence	—
										Glove usage	—		
	Healthcare workers	334|333		Primary	C,E,x	x				Handwashing frequency	↓	Skin condition	—
												HE prevalence	—
										Skin care usage	↑		
										Glove usage	—		
Moldovan et al. [43]	Healthcare workers	230	Prospective cohort study	Primary	x	x						Skin condition	—
												TEWL	↓
Oreskov et al. [44]	Hairdressers	43	Before‐after study	Primary	E		x			Contaminated skin area	↓		
Rasmussen et al. [45]	Production workers	503|396	Controlled before‐After study	Primary	x		x	x		Modified work	↑	CD incidence	↓
										Environment changes safety culture	↑		
Reich et al. [46]	Metal workers	131|172	Pretest‐posttest	Primary	S,P,E	x	x					CD prevalence	↓
Sedeh et al. [47]	Cleaners	100	Prospective cohort study (pilot)	Primary	x					Skin care and protection knowledge	↑		
Sell et al. [32, 33, 48, 49]^d^	Food or catering	380|285	Prospective cohort study	Primary	x			x		Glove usage	↑	Skin symptoms	↓
												CD prevalence	—
										Skin care usage	↑		
										Prevention knowledge	↑		
Soltanipoor et al. [50, 51, 52]	Healthcare workers	285|216	RCT	primary	C,P	x				Skin care usage	↑	HE prevalence	—
										Wet‐work activities	↓	HE severity	—
Symanzik et al. [53]	Healthcare workers	135|162	Nonrandomised comparative trial	Primary	C,P	x				Skin care usage	↑	HE prevalence	↓
												HE severity	↓
Trape et al. [54]	Healthcare workers	475	Pretest‐posttest	Primary	x		x			Latex‐free glove usage	↑	Skin condition	↑
Van der Meer et al. [55, 56, 57, 58, 59, 60, 61]^d^	Healthcare workers	876|773	RCT	Primary	P,E	x	x			Handwashing frequency	↓	HE prevalence	—
										Skin care usage	↑	Symptoms prevalence	—
												Skin condition	—
Wilke et al. [62]	Mixed	140|134	Prospective cohort study	Primary	S,C,P	x	x			Skin protection knowledge	↑		
										Skin knowledge	↑		
Winker et al. [63, 64]	Construction	198|287	RCT	Primary	x	x	x					TEWL	↓
												Skin condition	↑
Zack et al. [65]	Mixed	24	Qualitative study	Primary	x								
Zagrodney et al. [66]	Healthcare workers	254	Prospective cohort study	Primary	C,E	x				CD knowledge	↑		
										Skin care habits	↑		
										Handwashing frequency	↓		
Aktas et al. [67]	Hairdressers (student)	203	Non‐randomised comparative trial	Secondary	C,P				Reinforcement	Glove usage	↑	HE severity	↓
										Handwashing frequency	—		
Apfelbacher et al. [68]^d^	Mixed	253	Prospective cohort study	Secondary	S,C,P,E		x					Skin condition	↑
												Severity of skin lesions	↓
Arbogast et al. [69]	Mixed (fibreglass handling, automotive facility, petroleum worker, metal working)	336	RCT	Secondary	S,C	x						Skin condition	↑
												TEWL	↓
Attwa et al. [70]	Mechanics	47	Prospective cohort study	Secondary	S,C,P		x			CD knowledge	↑	Symptoms and signs of CD	↓
										Risk factors knowledge	↑		
										Prevention knowledge	↑		
Bauer et al. [71]	Food or catering	29	Prospective cohort study	Secondary	x	x	x			Efficacy with preventive measure	↑	Skin condition	↑
Berndt et al. [72]	Healthcare workers	25	RCT	Secondary			x					Skin condition	—
												Irritation	↓
												TEWL	—
	Healthcare workers	25		Secondary			x					Skin condition	—
												Irritation	↓
												TEWL	—
Boyle et al. [73]	Healthcare workers	50|25	RCT	Secondary			x					Skin condition	—
												TEWL	—
Brans et al. [74, 75]^d^	Mixed	231	Prospective cohort study	Secondary	S,C,P,E		x			Occupational footwear usage	↑	Symptoms food dermatoses	↓
										Occlusive shoes wearing duration	↓		
										Sock changing frequency	↑		
Dietz et al. [76]	Mixed	26|26	RCT	Secondary	S,P	x	x			Skin care usage	—	HE severity	↓
										Handwashing frequency	↓		
										Wet hands duration	↓		
										Occlusive gloves wearing duration	↓		
Filon et al. [77]	Mixed	54|48	RCT	Secondary	C,P,E	x	x			Handwashing frequency	—	Skin condition	↑
												TEWL	↑
										Prolonged glove usage	↓		
Fisker et al. [78, 79]	Mixed	376|380	RCT	Secondary	S,C,P	x	x	x		Skin care usage	↑	Skin condition	—
										PPE usage	↑	HE severity	↓
										Skin protection knowledge	↑		
Gasparini et al. [80]	Healthcare workers	28	Prospective cohort study (pilot)	Secondary	S,C,P,E		x			Risk factor knowledge	↑	CD prevalence	↓
										Skin care usage	↑		
Hansen et al. [81]^d^	Mixed	214	Pretest–posttest	Secondary	S,C,P		x			Prevention self‐efficacy	↑	Skin condition	↑
										CD knowledge	↑		
Heichel et al. [82]^d^	Healthcare workers	84	Prospective cohort study	Secondary	S,C,P,E		x					Skin condition	↑
		77		Secondary	S,C,P,E		x					Skin condition	↑
Held et al. [83]	Mixed	207|168	Prospective cohort study	Secondary	x	x	x			Wet hands duration	↓	Symptoms	↓
										Glove usage	↓		
										Handwashing frequency	—		
										Skin care usage	—		
Hoffmann et al. [84]^d^	Mixed	242	RCT	Secondary	x				SMS‐based aftercare	Skin care usage	—	Skin condition	—
										Skin self‐efficacy	—		
										Glove usage	—		
										Risk perception	—		
Ibler et al. [85, 86, 87]	Healthcare workers	123|132	RCT	Secondary	C, x	x	x			Skin protection knowledge	—	Skin condition	↑
										Handwashing frequency	—	CD severity	↓
										Skin care usage	—		
										Prolonged glove usage	—		
Kaatz et al. [88]	Food or catering	225	Prospective cohort study	Secondary					Communication tool	Intervention knowledge	↑		
Kugler et al. [89]^d^	Metal workers	128	Prospective cohort study	Secondary	S,C,P,E		x					CD severity	↓
Kutting et al. [90, 91, 92]^d^	Metal workers	1355	Retrospective cohort study	Secondary	S,C,P,E		x			Glove usage	↑	HE prevalence	↓
										Skin care usage	↑		
Kuwatsuka et al. [93]	Healthcare workers	16	Prospective cohort study (pilot)	Secondary	P	x	x					Skin condition	—
Mauro et al. [94]	Mixed	101	Prospective cohort study	Secondary	x		x					Skin condition	—
												TEWL	—
Mertin et al. [95]^d^	Metal workers	90	Prospective cohort study	Secondary	S,C,P,E		x			Barrier cream usage	↑	CD severity	↓
										Skin care usage	↑		
										CD knowledge	↑		
Nichol et al. [96]	Healthcare workers	220	Prospective cohort study	Secondary	x				Screening tool				
Nienhaus et al. [97, 98]^d^	Hairdressers	635	Prospective cohort study	Secondary	S,C,P,E		x			Glove usage	↑	CD severity	↓
										Skin care usage	↑		
										CD knowledge	↑		
Schuler et al. [99]^d^	Mixed	502	Prospective cohort study	Secondary	S,C,P,E		x			Skin care usage	↑	Skin irritation	↓
										Glove usage	↑	Skin symptoms	↓
										Prevention self‐efficacy	↑		
Schürer et al. [100]^d^	Healthcare workers	209	Prospective cohort study	Secondary	S,C,P,E		x					TEWL	↓
												Skin condition	↑
Shahraki et al. [101]	Healthcare workers	9|9	RCT (pilot)	Secondary			x			Wearing cotton‐polyester gloves below gloves	↑	Skin symptoms	—
Soder et al. [102]^d^	Food and catering	212	Prospective cohort study	Secondary	S,C,P,E		x			Skin care usage	↑	CD severity	↓
										Handwashing frequency	↓	CD prevalence	—
										Glove usage	—		
Weisshaar et al. [103, 104]^d^	Healthcare workers	311	Prospective cohort study	Secondary	S,C,P,E		x			Glove usage	↑	CD prevalence	—
										Skin care usage	↑		
Weistenhofer et al. [105]	Production workers	270|135	Retrospective cohort study	Secondary	x		x					Skin condition	↑
												TEWL	↓
Wilke et al. [106]^d^	Mixed	134	Prospective cohort study	Secondary	S,C,P,E		x			Handwashing frequency	↓	HE severity	↓
										Skin care usage	↑		
Wilke et al. [107]^d^	Mixed	105	Prospective cohort study	Secondary	S,C,P,E		x			CD knowledge	↑		
										Protection knowledge	↑		
Wilke et al. [108]^d^	Metal workers	178	Prospective cohort study	Secondary	S,C,P,E		x			Skin management self‐efficacy	↑	CD severity	↓
												HE prevalence	↓
Skudlik et al. [109, 110, 111, 112, 113, 114, 115, 116]^d^	Mixed	1788	Prospective cohort study	Tertiary	S,C,P,E	x	x			Hand washing frequency	↓	HE severity	↓
										Hand disinfection frequency	↓	Return to work	↑
										Skin protection creams usage	↑		
										Emollients usage	↑		
										Protective gloves usage	↓		
Breurer et al. [117]^d^	Mixed	122	Pretest – posttest	Tertiary	S,C,P,E	x	x			Skin management self‐efficacy	↑	CD severity	↓
										Skin protection knowledge	↑	CD symptoms	↓
Chen et al. [118]^d^	Healthcare workers	18	Prospective cohort study	Tertiary	x			x		Modified duties	↑		
										Return to patient care	↑		
										PPE usage	—		
										Skin care usage	—		
										Skin protection knowledge	↑		
Gill et al. [119]	Mixed	42 patients 10 dermatologists	Feasibility study	Tertiary			x		Teledermatological consult				
Holness et al. [120]	Mixed: Automotive workers	30	Retrospective cohort study	Tertiary	x	x		x		Modified tasks	↑	Return to work	↑
	Healthcare workers	21											
	Manufacturing workers	62											
	Services industry	19											
Kudla et al. [121]^d^	Mixed	28|22	Prospective cohort study	Tertiary				x	Communication tool	Modified duties	↑		
Loi et al. [122]^d^	Healthcare workers	21	Prospective cohort study	Tertiary	C,E	x		x		Modified duties	↑	Skin condition	↑
Matterne et al. [123]^d^	Mixed	102	Prospective cohort study	Tertiary	S,C,P,E	x	x			Skin management self‐efficacy	↑		
										Skin protection behaviour	↑		
Mollerup et al. [124]	Mixed	142|150	RCT	Tertiary	x				Digital counselling			Symptoms	↓
												Burden of disease	↓
												CD severity	—
Ristow et al. [125]^d^	Mixed	72	Design article	Tertiary					App‐based maintenance				
Skudlik et al. [126]^d^	Metal workers	59	Prospective cohort study	Tertiary	S,P,E	x	x			Skin knowledge	↑	CD prevalence	↓
										Skin protection knowledge	↑		
Skudlik et al. [127]^d^	Mixed	1486	Prospective cohort study	Tertiary	S,P,E	x	x			Skin protection behaviour	↑		
										Job retention	↑		
Brans et al. [128]^d^	Metal workers	114	Prospective cohort study	Secondary	S,C,P,E		x			CD knowledge	↑	Symptoms severity	↓
										Extended wet work	↓		
										Glove usage	↑		
										Handwashing frequency	↓		
		83		Tertiary	S,C,P,E	x	x					CD severity	↓
Schwanitz et al. [129]	Hairdressers	73|112	Retrospective cohort study	Primary	P	x	x					Skin condition	—
	Hairdressers	70		Secondary	P	x	x			Skin protection behaviour	↑	Skin condition	—
	Mixed	155		Tertiary	P	x	x			Glove usage	↑	Skin condition	—
										Skin protection behaviour	↑		

^a^ Number of participants (N). If a control group was present, the ‘|’ symbol separates N intervention and N control.

^b^ It is stated if topics discussed are known to include Skin health (S), Skin Care usage (C), PPE usage (P) or Exposure reduction (E).

^c^ Trend displayed as reported by the authors, where ↑ indicates the outcome had increased, ↓ the outcome had reduced, and—when there is no to minimal change. For negative outcomes, a reduction constitutes a decrease in the negative effect (e.g., a decrease in severity of contact dermatitis (CD) constitutes less severe symptoms of CD).

^d^ Included (in part) during analysis on implementation strategies and outcomes (see Table 2).

### 3.3 Preventive Interventions

Out of the 79 included studies, 31 studies were aimed at primary prevention, 34 studies were aimed at secondary prevention and 12 studies were aimed at tertiary prevention (Table 1). Furthermore, two studies included two or more prevention levels [128, 129].

Four main preventive measures were identified: education, skin care, use of personal protective equipment (PPE) and workplace adaptations. In the majority of studies, a combination of these measures was implemented and studied. Most often this involved education combined with another preventive measure.

Educational interventions were reported in 69 studies (87.3%), providing (a combination of) information on the skin in general (*n* = 31), use of skin care products (*n* = 37), use of PPE (*n* = 39) and reducing exposure (*n* = 33). In 46 studies, education on skin care products or PPE was combined with the distribution of products. The method of delivering the education varied from group lectures, individual feedback and workshops to distribution of informational materials.

Provision of skin care, such as emollients or moisturisers, as part of the intervention was reported in 31 studies (39.2%). In the majority of cases these were distributed to the participants individually; however, in three studies the skin care products were made available at the workplace site, either through dispensers or by distribution at the workplace [41, 55, 122].

PPE provision was reported in 52 studies (65.8%) and included the provision of barrier creams and gloves. Three studies solely provided PPE [38, 72, 73], while others combined this with other intervention components.

Workplace adaptations occurred in 10 studies (12.7%) and focused on reducing exposure to risk factors. These were either through modifying work tasks (such as less frequent contact with the irritants or allergens, or by reducing the exposure to wet work), or by making adaptations in the used products at the workplace.

Other preventive measures that were explored included screening tools and communication tools [88, 96, 121], skin checks [23, 27, 125] and reinforcement of skin barrier by UV‐B hardening [20].

### 3.4 Behavioural and Clinical Outcomes

Of the 79 included studies, 58 reported behavioural and knowledge outcomes and 60 clinical outcomes, with 44 reporting both; five studies reported neither, consistent with the study design (e.g., intervention development). The mixed study designs included interventions with and without control groups.

In the context of primary prevention (31 studies), knowledge improved over time in all 11 studies that assessed it. PPE usage was assessed in 13 studies showing improvement over time in 8, and skin care usage was assessed in 13, also showing improvement over time in 8. Handwashing frequency was reported in 8, with mixed results and work modifications in 4 showing increased modified duties in all 4 studies.

In secondary prevention (34 studies), knowledge (*n* = 12), PPE usage (*n* = 11), skin care practices (*n* = 11) and work modifications (*n* = 3) were assessed. Two‐thirds of these studies showed improvement over time in these outcomes. Handwashing frequency (*n* = 7) showed mixed results in the studies that included this outcome.

In tertiary prevention (12 studies), knowledge (*n* = 6) and work modifications (*n* = 4) were evaluated more frequently than PPE usage (*n* = 2), skin care usage (*n* = 2) and handwashing frequency (*n* = 1). Knowledge improved over time in all studies that assessed it. Similarly, work modifications and handwashing frequency saw a positive trend. PPE and skin care usage were reported to improve in one of the two studies assessing these outcomes.

Clinical outcomes included OCD or hand eczema (HE) prevalence and skin condition (i.e., severity, symptoms and skin barrier function). Across primary prevention, 14 studies assessed OCD or HE prevalence. These were assessed compared to a control group in nine, with six showing a decrease in prevalence. These ranged from smaller differences during the intervention (e.g., an 8.8% increase in the control group [53]) to larger differences between intervention and control groups (e.g., a 3‐year prevalence of 39.0% versus 16.0% [46]). The other three studies reported minor to no change in prevalence between intervention and control group, according to authors. Skin condition improved in four studies, showed no change in four and worsened in one study [40]. Severity of CD decreased in two studies and saw minimal change in one study [50].

In secondary prevention (34 studies), five studies assessed OCD or HE prevalence. These were assessed compared to a control group in two studies. One of these showed a decrease over time, from 56.2% to 41.0% [31], with the other reporting smaller differences between control and intervention group (15.5% to 13.0%, respectively) [90]. Skin condition improved in nine studies, but authors reported minor to no change in six studies. Severity of the skin condition decreased in all 10 studies that assessed it.

In tertiary prevention (12 studies), where all participants suffer from OCD, one single‐arm study showed a 52.5% decrease in OCD prevalence over time [126]. Skin condition was an outcome in one study, showing improvement over time [122]. Two out of three studies assessing the severity of CD reported a decrease over time.

### 3.5 Implementation Strategies

Eleven programs researched and reported at least one implementation outcome of their strategies (Table 2). In nine of these programs, multiple implementation strategies were employed [27, 32, 42, 55, 68, 83, 96, 118, 122]. Two programs focused on communication through a single implementation strategy [47, 121]. Three programs conducted in‐depth evaluations of implementation outcomes in separate articles, using process evaluations or narrative summaries of the implementation process [32, 33, 56, 57, 68, 109]. Implementation strategies targeted multiple levels. At the individual level, workers received group or individual education and feedback or were tasked with acting as role models. At the organisational level, skin care guidelines were introduced. Management‐level strategies included the establishment of working groups to support implementation. The Hands4U program [55], the program on Danish wet‐workers [32] and the program in German nursing homes [83] engaged workers and managers in participatory teams as part of their implementation strategy.

**TABLE 2:** Implementation strategies and outcomes.

References	Implementation strategy	Acceptability	Adoption	Appropriateness	Feasibility	Fidelity	Implementation costs	Penetration	Sustainability
Skudlik et al. [68, 74, 75, 81, 84, 89, 90, 91, 92, 95, 97, 98, 99, 100, 102, 103, 104, 106, 107, 108, 109, 110, 111, 112, 113, 114, 115, 116, 117, 123, 125, 126, 127, 128]	Group education seminars Individual advice protective equipment Dermatological consult	A significant majority reported high satisfaction with the seminars; Metalworkers 96.4% of found attending worthwhile [108]. 98.8% were satisfied with the atmosphere and content delivery [81]. 94.1% were satisfied with the overall organisation before the seminar [81]. 92.3% rated it as helpful [68] 83.9% rated the seminar as beneficial [102] 81.1% rated as successful/very successful by the evaluating dermatologist [89]	No information	The interventions were seen as helpful and beneficial by the majority of participants Individual advice of protective equipment was tailored to the profession Participants reported better experience with GLs vs. previous gloves [82] Similarly, in cleaning and kitchens employees 83.9% [102] and in metalworkers 83% [108] considered their participation beneficial	No information	*The program is part of the German Statutory Accident Insurance institutions and is only followed in certain centres within the country. Here the protocol is followed* The prevention program is standardised across the centres and described consistently across articles, suggesting that delivery was likely uniform, although this cannot be verified	The intervention is free of charge for participants as it is funded by the German Statutory Accident Insurance institutions (e.g., BGW, BGHM)	High as the German Statutory Accident Insurance implemented this into practice. For companies it is mandatory to be part of the German Statutory Insurance	Sustainability is high as the SIP and TIP have been running since 2003 and has been part of the standard practice since 2005
Hoffmann et al. [84]	SMS‐based aftercare	SMS were well accepted and easy to follow	No information	Content seen as relevant and applicable to work context (87.5%) – 97.5%: SMS was easy to understand – 97.5%: length was appropriate – 90.0%: number of SMS appropriate	5×/week SMS delivery was sustained	No information	No information	No information	No information
Van der Meer et al. [55, 56, 57, 58, 59, 60, 61]	Participatory working groups Role models Group education sessions Reinforcement through leaflets and posters	Working groups included managers and found the guidance, content of the working group, and role model role satisfactory (all rated 4.0 or higher on 1–5 range) Satisfaction within employees was above the midpoint for all components	Most recommendations were in line with the company's health protocol 97.4% of participants noticed their department participated in elements of the study	Using hand disinfectant or hand hygiene, use of gloves during wet work, use of moisturiser, removing jewellery and reducing wet were all deemed appropriate measures Use of cotton under gloves was deemed appropriate except while performing delicate tasks Overall the intervention was a good fit, although there were doubts concerning the role models element	The strategy was feasible in a healthcare setting, though the role model component warrants further investigation	For the working groups the fidelity of occupational nurses was 84.5% For role models less than 40% of participants interacted with role models For education settings only half the workers attended For reinforcement 84.6% of workers noticed the information	The multifaceted implementation strategy was not cost‐effective in comparison with the control group from both a societal and employers' perspective, nor did it lead to a positive financial return for the employer	Out of 104 solutions conceived by the working group, 87 were realised	No information
Loi et al. [122]	Group education Substitution of used products Modified work tasks Involvement employer (medical letters)	Participants reported that their workplace supervisors were accommodating to the interventions by supplying the proposed hand hygiene products and making adjustments to temporarily reduce clinical duties (supervisor level) Alcohol‐based hand rub received positive feedback from workers, who considered it a gentler alternative (individual level)	Four out of six possible effective workplace adaptations were implemented	The study had elements focussed on irritant and allergic contact dermatitis and tailored interventions accordingly The participants reported that their workplace supervisors were accommodating to the interventions (individual level)	No information	Workplace interventions were implemented for all participants, except temporary job modifications which was only done for those with hand dermatitis All participants were given medical letters detailing the recommendations to pass to their superiors The participants reported that their workplace supervisors were accommodating by supplying the participants with the proposed products and making adjustments their clinical duties	No information	No information	No information
Mygind et al. [31, 32, 33, 48, 49]	Establishment and education of a project group of safety representatives (including managers) Resource persons (role models or change agents) Establishing occupational health management system	In interviews the management of 2 out of 6 gut cleaner departments explicitly mentioned approving the project. No information was available for the other 4 (organisational level) Employees in 4 out of 6 departments of the gut cleaners explicitly mentioned their satisfaction or enthusiasm about the project or elements of the project (individual level)	The company's willingness to fund most expenses indicates strong motivation to support and implement the interventions 6 out of 6 departments joined the skin project in gut cleaners (organisational level) 3 out of 6 departments mentioned uncooperative managers or other employees in terms of adoption of the project (individual level)	In general discussing prevention among employees by the workforce increased where the intervention was received In gut cleaners: 2 out of 6 departments explicitly mentioned satisfaction with the content of the project (individual level)	1 out of 6 departments had to shut down the project during the study period	Fidelity of the implementation strategies were rated on a 0 to 3 score across the 6 departments in the gut cleaners Safety representatives had a mean rating of 1.81 (range 1.27–2.36) Resource persons a mean of 1.86 (range 1.32–2.32) The management system had a mean rating of 1.80 (range 1.13 tot 2.38)	No information	In both gut cleaners the amount of people that received information and the degree to which workplace exposure was discussed increased In dairy farmers it also increased significantly from 41.5% to 62.6% (*n* = 296) The intervention was rolled out in three industries at the same time	No information
Chen et al. [118]	Communication with stakeholders by a return to work coordinator (RTWC) Identify concerns and barriers by RTWC Formal written RTW plan, monitored by RTWC	There was good communication between all parties in 17 cases (94%)	A graduated RTW plan was formulated and customised for all nurses (organisational level) All 18 nurses referred to and enrolled in the RTW program (individual level)	7 nurses (39%) had ongoing symptoms that required different treatment Five nurses (28%) reported continued exposure at work to either high levels of irritation or their specific allergens Two nurses (11%) found the skin care management cumbersome and interfered with their duties	For 17 nurses (94%) modified work was available A graduated RTW plan was formulated and customised for all nurses	All nurses (100%) adhered to skin management and return to work protocol Workplace modifications could not be fully implemented. In 2 cases (11%) of the recommendations for hand sanitizers were rejected by Infection Prevention and Control departments	No information	No information	No information
Held et al. [83]	Group education for participatory team Educational program for a group of 10–20 employees (including management, local safety board and employee from sector) Communication of information to colleagues by participatory team members Skin care policy including written instructions Free provision of cotton gloves and moisturisers	No information	No information	59% of participants indicated they benefitted hugely from the program	No information	97% of the employees had received moisturisers and 79% cotton gloves. 90% of the participants indicated receiving information about good skin care during intervention	No information	No information	No information
Madan et al. [42] (Madan, 2019 #3910)	Implementation plans were developed by nurses Skin care was provided both in the workplace, as well as for personal usage Educational material was distributed to all staff	No information	A total of 54 sites were assessed for eligibility for the study. Of these, 35 sites agreed to participate and 19 were not able to participate. Of the 35 participating sites, five recruited student nurses only, 18 recruited Intensive Care Unit (ICU)/Special‐Care Baby Unit (SCBU) nurses only and 12 recruited both student and ICU/SCBU nurses	90% of nurses agreed or strongly agreed that the information was easy to understand, it was sufficiently detailed, was relevant and was presented clearly and that the intervention itself was easy to use	No information	39% of the student nurses and 48% of the ICU/SCBU nurses in the intervention plus arm reported that they had accessed the BCP intervention during the study	The Behaviour Change Package is considered cost‐effective from a health‐care perspective if only looking at reducing hand dermatitis	The study identified all NHS sites in the UK that train nurses and have an in‐house occupational health service and at least one ICU	No information
Brown et al. [27]	Skin care policy Provision of information Distribution of gloves and cream Skin checks for workers	All interventions were found to be acceptable to some extent Skin care policy envisaged by senior management Provision of information: no information Gloves and cream: Workers' said a similar service or information should be provided for all workers Skin checks: Employees at both companies believed skin checks would be a positive move and the company should provide it if requested but were unsure if it changed working practice	Not many companies were willing to participate	Skin care policy: no information Provision of information: General consensus that more information should be introduced, especially with regards to long‐term health effects Gloves and cream: Print workers said that it was helpful to receive the advice of an expert All those interviewed agreed that the information could potentially have a beneficial effect Skin checks: not all workers saw reason for following advice, as they ‘saw no reason for change’ Skin checks: Employees were unsure if skin checks changed working practice	No information	Skin care policy: mixed success, due to time scale and other priorities Conflicting views on information dissemination and communication; 55% of the workers reported that all information had been passed on to them; 2 out of 3 managers had seen the information	‘Low‐cost’	No information	No information
Kudla et al. [121]	Development of communication tool with workers and employee representatives Completion of a workplace prescription (WP) by the dermatologist, consecutively shared by workers with the employer Education through website Reinforcement through email	There was consistency in workers' views (for content and style) that the WP tool would be useful	9 (41%) provided the WP to their employer and 8 (36%) provided only verbal results	For those that provided the WP, 7 reported the recommendations were received positively, 1 reported that the supervisor was uninterested and 1 reported a negative response (too many restrictions)	No information	No information	No information	No information	No information
Nichol et al. [96]	Screening tool provision to workers by researchers, combined with information sheet Encouragement for a follow‐up to primary care after positive screening	99.5% of participants found the tool easy to use	No information	84% of participants found screening for hand dermatitis very important	93% of participants reported that using the tool took less than 2 min to complete	No information	No information	The Hand Dermatitis Screening Tool was also used in the acute health care sector	No information

*Note:* Overview of reported program outcomes related to implementation. Information on the eight implementation outcomes is presented as described in the original papers and summarised where necessary for clarity. Absence of reported information is indicated accordingly.

Out of these programs, five were conducted in mixed occupational populations [32, 68, 83, 84, 121], five in healthcare workers [42, 55, 96, 118, 122] and one in the printing industry [27]. Role models were applied both in mixed populations [32] and among healthcare workers [55], highlighting recurring implementation strategies across different occupational settings.

The most commonly researched implementation outcomes were acceptability and appropriateness, which were typically assessed through user feedback, either during the initial development of the intervention or after its implementation.

*Acceptability* was reported for 10 programs. The majority of programs researched this through interviews or other qualitative methods during and after the study, and two programs did quantitative assessment through rating the satisfaction with a score [57, 68]. At the organisational level, employers or managers were asked in 4 out of 10 programs to comment on their perspective on acceptability of the intervention program. In 9 out of 10 programs, acceptability by the employees was addressed. In general, programs reported good acceptability by both employers and employees, both in the multicomponent and education‐only intervention programs. In some programs, comments were found on employers or employees who did not find the program acceptable. For example, employees found following the program cumbersome [118].

*Adoption* was reported in six programs, all of which featured a multifaceted approach. Four out of these six referred to the amount of companies or departments that were approached and subsequently involved in the study. For instance, the Behavioural Change Package (BCP) in the United Kingdom (UK) saw that out of 54 eligible National Health Service (NHS) locations, 35 adopted the program [42]. One study reported on six possible measures adopted four of these measures [122]. Finally, one study reported on adoption at individual level, where the Return To Work (RTW) program had all 18 nurses that were referred adopt the program [118]. Generally, researchers reported on their recruitment, and little information is available on the sites that did not adopt the program.

*Appropriateness* was evaluated in all programs, primarily from the perspective of stakeholders. This assessment was conducted through interviews and other qualitative methods, both during and following program implementation. Overall, participants expressed positive views regarding program appropriateness, particularly with respect to the alignment of intervention components with job‐specific activities. Such evaluations were most frequently observed in primary and secondary prevention programs. The tertiary prevention programs by Chen et al. and Loi et al. examined appropriateness from the perspective of supervisors, focusing on their willingness to implement RTW measures [118, 122].

*Feasibility* was addressed in five programs. Three primary and secondary prevention programs conducted feasibility assessments as part of their research plan [42, 55, 68]. Feasibility of the program was generally rated positively, although specific elements, such as role models [55], were questioned. The level of detail reported on feasibility was limited for most programs. In the tertiary prevention programs [118, 122], feasibility was assessed retrospectively, focusing on challenges encountered during the study period.

*Fidelity* was reported in eight programs, referring to the extent to which participating entities adhered to the intended protocol. This was most commonly assessed in programs with multiple components or a multifaceted approach, where individual elements could be evaluated separately. For example, in Van der Meer's study, fidelity was examined in relation to the working groups or ‘Dermacoaches’, who served as peer role models [57]. While the fidelity of the group as a whole could not be determined for the overall intervention, more than 80% of the working group members followed the protocol. In primary and secondary prevention programs, fidelity primarily concerned participant adherence to intervention components. In tertiary programs, the main fidelity challenge related to the feasibility of implementing certain modified work duties.

*Implementation costs* were reported in three programs [41, 56, 109], as these did an economic evaluation of the interventional program in their setting. The BCP in the UK reported the material components cost an average of £13 per Intensive‐Care Unit nurse and £14 per student nurse [41]. The Hands4U study reported that their multicomponent prevention program, including material and implementation, costs €113.84 per worker (price point 2010) [56]. The Tertiary Individual Prevention program in Germany costs an average of €5996.30 per person, including care and materials [109].

*Penetration* was reported in five programs, all of which involved multicomponent interventions. Three programs [32, 42, 83] described program reach in terms of the number or diversity of providers, while the others reported on program integration within existing structures. The programs in Danish wet‐work occupations [32] and the German strategy for Secondary and Tertiary Individual Prevention [68] demonstrated the implementation of similar strategies across diverse occupational groups. Although these programs incorporated individual feedback and employee involvement, their core concepts remained consistent across target audiences.

None of the included programs explicitly investigated the *sustainability* of program implementation. While some programs suggest that certain preventive measures might be sustainable [55], no follow‐up beyond the study period was conducted to assess the long‐term sustainability of their implementation. Notably, the German national Secondary and Tertiary Individual Prevention program is an example of long‐term applicability. This multi‐step, interdisciplinary intervention has been implemented across various high‐risk occupational groups, including cleaners, metalworkers, hairdressers and healthcare workers. The program is embedded within secondary individual prevention frameworks and is delivered by multiprofessional teams. With its national rollout and sustained implementation over more than 10 years, the program offers indirect evidence of sustainability, even though no studies explicitly addressed this aspect.

## 4 Discussion

### 4.1 Main Findings

This scoping review identified a range of organisational and individual interventions to prevent (severity of) occupational contact dermatitis consisting of (combinations of) education, personal protective equipment, skin care and workplace adaptations. Most studies reported on behavioural and clinical outcomes. Behavioural outcomes generally showed improvements in the use of PPE and skin care practices. Clinical outcomes, assessed through self‐reported data and dermatological indices, showed a trend towards reduced OCD prevalence and improved skin condition, though findings from randomised controlled trials were inconsistent. While the content and outcomes of preventive interventions were sufficiently reported, only a small proportion of studies evaluated implementation strategies, especially at organisation and management level. These studies reported generally positive findings regarding acceptability and appropriateness, while feasibility, adoption, fidelity, costs and penetration were less assessed. No studies explicitly evaluated sustainability directly.

### 4.2 Interpretation of Findings

The studies included occupations with an increased risk for OCD, such as healthcare workers, hairdressers, construction workers, metal workers and cleaners [130]. These occupations are characterised by frequent exposure to wet work, irritants and allergens. Most preventive interventions at the individual level, such as improving the skin care and reducing wet work exposure, aligned with existing behavioural change models [131, 132, 133]. While these studies applied similar intervention components, their effectiveness varied, at least partly due to differences in implementation strategies.

The observed variation in implementation strategies across countries and target populations can be partly explained by contextual factors such as working conditions, regulatory frameworks and occupational health policies. For example, Germany's established protocols for managing OCD have shaped interventions that differ in type and focus from those implemented in other countries [5]. This highlights that in practice, it is not only the preventive measures themselves that should be adapted to the target population, but also the core implementation strategies. Common strategies, such as educational programs or provision of feedback, often need to be tailored to the specific needs, preferences and context of the workforce to ensure successful adoption and uptake, which is a central principle in implementation science [134].

Preventive interventions for OCD are ideally guided by hierarchical principles like the STOP principle, prioritising substitution, followed by technical, organisational and personal measures [135]. In some occupations, such as in healthcare, construction, metal industry, food industry or hairdressing, substitution or technical measures are not always feasible due to the nature of the work tasks. This principle highlights the importance of implementing these strategies in the recommended hierarchical order, first addressing organisational‐level measures such as workplace adaptations and guidelines and then supplementing them with personal‐level measures including education, PPE and skin care routines.

There is no implementation framework specifically for OCD, but frameworks such as the Consolidated Framework for Implementation Research (CFIR) [10] can provide insight in understanding why preventive measures are or are not successfully implemented in practice. These frameworks guide the analysis of intervention characteristics, outer setting (e.g., legislation), inner setting (e.g., the organisation), the characteristics of individuals (e.g., knowledge, attitudes) and the implementation process, and can help to select suitable implementation strategies. In most identified studies little information was available on the rationale for selecting specific implementation strategies, that is, what specific barriers and facilitators the strategy focused on, and both if and how the occupational context played a role. This information gap obstructs a clear interpretation of the information found on implementation strategies and their outcomes: when we do not know what problem the implementation strategy was needed for, we cannot conclude whether it was successful or not. For example, when adoption using a particular strategy was found to be high, it does not necessarily mean that this can be attributed to the strategy. It might not have been a problem in the first place for that specific occupational group or in that specific company.

### 4.3 Implications for Research

We observed in this scoping review that assessing implementation effectiveness of preventive interventions for OCD remains challenging. In general, there appears to be more emphasis on intervention effect outcomes in terms of behaviour or skin condition rather than the contextual factors that contribute to their success. Addressing implementation strategies, it is warranted to understand how implementation processes contribute to the prevention of OCD. Ideally, research should inform the sustainability of interventions, examining which implementation strategies are most effective, how they interact with different occupational contexts and how barriers and facilitators shape uptake and fidelity. To date, the evidence does not provide such guidance. When assessing the findings of this review in more detail, we see different implementation strategies and different combinations of implementation strategies, but no direct comparisons of strategies. It would be valuable for future research to shed more light on the effects of implementation strategies across different strategies, occupations and contexts.

### 4.4 Implications for Practice

The implementation strategies we identified appear broadly applicable across various settings. In the prevention of OCD many different stakeholders are involved: workers, employers, works council, occupational health care, occupational hygienists, primary care, dermatologists, allergists and insurers. It is challenging to streamline the co‐operation between those stakeholders for the purpose of tackling OCD in a company or industrial sector. Our scoping review identified a couple of best practices [32, 42, 55, 68, 127] and these provide a starting point for designing implementing strategies for OCD prevention programs. At the individual level, practical actions could include job‐specific information with suitable PPE and skin care [55, 68], as well as improving communication of (dermatological) advice by giving relevant tools to stakeholders improving case management [118, 121]. At the organisational level, efforts might focus on improving access to protective measures in the workplace [42, 55], offering continuous training [32] and integrating skin health monitoring into routine procedures [96].

### 4.5 Strengths and Limitations

This review is the first to include both observational studies and controlled trials across all types of organisational and individual preventive measures for OCD. Their inclusion offers valuable insights into the range of preventive measures and their implementation outcomes in diverse settings. A significant challenge was that many researchers conducting intervention studies did not anticipate or design their research to be evaluated based on implementation outcomes. Consequently, implementation processes were often either not reported or only briefly mentioned in the majority of studies. Furthermore, although our search across multiple databases offers a comprehensive overview, legislation and policy‐level interventions, as well as treatment‐focused studies, were excluded, as they fall beyond the scope of preventive strategies at the individual and organisational levels. Nevertheless, the findings provide valuable insights for both individual healthcare professionals and collective workplace initiatives aimed at preventing OCD. A final limitation of this review is the restriction to studies published in languages understood by the researchers, which may have led to an overrepresentation of studies conducted in Western Europe. This is unlikely to have substantially influenced the outcomes, as the core preventive measures and implementation strategies are comparable across settings and most relevant studies are published in English.

## 5 Conclusions

In conclusion, preventive interventions for occupational contact dermatitis consistently focus on education, personal protective equipment, skin care and workplace adaptations. Implementation strategies for these preventive interventions were described for 38% of the included studies, primarily focusing on acceptability and appropriateness and to a much lesser extent on adoption, feasibility, fidelity, cost, penetration and sustainability. Evaluation of implementation is limited, despite it being essential to ensure preventive interventions are delivered efficiently. Well‐designed implementation studies can guide transparent, evidence‐based and effective prevention efforts.

## Author Contributions

**Jonathan A. G. Jonker:** conceptualisation, writing original draft, methodology, data curation and analysis, project administration. **Sietske J. Tamminga:** conceptualisation, methodology, data curation, supervision, writing. **Felicia S. Los:** conceptualisation, data curation. **Parel M. V. Janse:** data curation. **Sanja Kezic:** conceptualisation, methodology, supervision, writing. **Henk F. van der Molen:** funding acquisition, conceptualisation, methodology, supervision, writing. **Julitta S. Boschman:** conceptualisation, methodology, data curation, supervision, writing. All authors contributed to reviewing the manuscript.

## Funding

This work was supported by the Netherlands Expertise Centre for Substance‐Related Occupational Diseases: Lexces, as part of a government‐subsidised research initiative aimed at the prevention of occupational diseases.

## Conflicts of Interest

The authors declare no conflicts of interest.

## Supporting information


**Supplementary File 1.** Research protocol.

**Supplementary File 2.** Table on study characteristics.
**Table S1:** Characteristics of the included studies.

**Supplementary File 3.** Overview of Data Items.

## Data Availability

The data that support the findings of this study are available from the corresponding author upon reasonable request.
