# Supplementary material for: Interventions and Implementation Strategies for Preventing Occupational Contact Dermatitis: A Scoping Review
Source: Contact Dermatitis. 2026 Feb 15;94(5):437–64. doi: 10.1111/cod.70113 (PMC13070721; doi:10.1111/cod.70113)
Supplement: Supplementary file 1 — Research protocol. [file COD-94-437-s003.docx]

**Article name:** Interventions for preventing occupational contact dermatitis and their implementation: a scoping review

**Journal name:** Contact Dermatitis

**Authors’ information:** Jonathan A.G. Jonker^12^*, Sietske J. Tamminga^12^, Felicia S. Los^12^, Parel M.V. Janse^12^, Sanja Kezic^12^ , Henk F. van der Molen^12^, Julitta S. Boschman^12^

^1^Amsterdam UMC location University of Amsterdam, Public and Occupational Health, Meibergdreef 9, Amsterdam, The Netherlands

^2^Amsterdam Public Health Research Institute

(*Corresponding author: j.a.g.jonker@amsterdamumc.nl)

**Supplementary file 1. Research protocol.**

The review protocol has also been registered and published in the Open Science Framework (OSF), accessible through osf.io/9gvab.

# Title

Interventions for preventing occupational contact dermatitis and their implementation: a scoping review protocol

# Authors

# Jonathan A.G. Jonker^12^, Sietske J. Tamminga^12^, Felicia S. Los^12^, Sanja Kezic^12^, Henk F. van der Molen^12^, Julitta S. Boschman^12^ ^1^Amsterdam UMC, Department of Public and Occupational Health, Amsterdam, The Netherlands ^2^Amsterdam Public Health Research Institute

# Research question

This review aims to provide broad insight into interventions and implementation strategies aimed at primary, secondary or tertiary prevention of occupational contact dermatitis.

As such the research questions are:

1. Which interventions aimed to prevent Occupational Contact Dermatitis (OCD) have been developed and evaluated?
2. What is the performance of implementation strategies for these OCD interventions?

This review is being conducted in the scope of a larger project aimed at the development and implementation of a Workers’ Health Surveillance (WHS) module to identify and prevent OCD in the Netherlands.

# Inclusion criteria

Included studies:

- Target workers or apprentices as studied population
- Target occupational contact dermatitis
- Describe one or more interventions specific to contact dermatitis that are connected to the workplace or a component of the intervention occurred at the workplace
- Present original study data OR are a systematic review (at least 2 databases, duplicate and quality assessment)
- Have full text available in English, Dutch, German or French
- Are published in or after 2000

Table 1 | Inclusion and exclusion criteria.

| Included studies | Excluded studies |
| --- | --- |
| **Population** | |
| Target:   - Workers (employees or self-employed, fulltime or parttime) - Apprentices (interns, students, or involved in practice in some way) | Target:   - General population - Mix of general population and workers - Work-status unclear |
| **Intervention/Exposure** | |
| - Targets occupational contact dermatitis - Describes one or more interventions, a multi-component intervention, strategy, or preventative measures specific to contact dermatitis (CD) - Describes intervention(s) connected to the workplace or a component of the intervention occurred at the workplace. | - Solely promote general well-being (nutrition, smoking cessation) in relation to OCD - Solely promote mental well-being in relation to OCD - Target skin cancer, other skin problems or skin problems not further defined |
| **Comparator / Context** | |
| Not applicable | Not applicable |
| **Outcome** | |
| Not applicable | - Report on (changes in) legislation |
| **Study Characteristics** | |
| - Presents original study data from:   - Experimental studies (i.e. randomized controlled trials [RCTs], cluster-RCTs or non-randomized/quasi experimental trials)   - Observational studies (cohort, cross-sectional, case-control, interrupted time series)   - Qualitative studies (i.e. describing barriers and facilitators for an existing or proposed intervention/strategy) - Systematic review (at least 2 databases, duplicate and quality assessment) to identify eligible original studies - Study protocol to identify full original studies | - Does not concern an intervention or strategy/advice or follow-up - Concerns a clinical trial of a treatment option (i.e. comparisons of different types of medication or medication vs placebo/no treatment) to evaluate or test medical interventions. - Consist only of summaries, commentaries or conference abstracts - Has the wrong article type (Case studies, narrative review articles) |
| **Other** | |
| - Full text available in English, Dutch, German or French - Published in or after 2000 | - Full text not available in English, Dutch, German or French - Published before 2000 |

Table 2 | Key components for structuring the search strategy and selection criteria derived from the PICO (population, intervention, control/comparator and outcomes) framework.

| **Definition** | |
| --- | --- |
| Population | Workers or apprentices exposed to irritants or allergens in the workplace |
| Intervention | Workplace or individual modifications to prevent occupational contact dermatitis |
| Control | Not applicable |
| Outcome | Not applicable |

# Methods

The proposed scoping review will be conducted using the Joanna Briggs Institute (JBI) methodological framework for scoping reviews [1].

# Search Strategy

A three-step search strategy will be utilized in this review. First an initial limited search of MEDLINE (PubMed) was undertaken to identify articles on the topic. The text words contained in the titles and abstracts of relevant articles, and the index terms used to describe the articles were used to develop a full search strategy for MEDLINE, Embase, Cochrane CDSR, CENTRAL and CINAHL (see Appendix I). The search strategy, including all identified keywords and index terms, was adapted for each included database. Systematic reviews identified through the search that are deemed relevant to the research question will be screened for additional studies. Studies published in English, Dutch, German or French and published in or after January 2000 will be included.

# Study/Source of evidence selection

Following the search, all identified citations will be collated into one Endnote library and duplicates removed through dedupendnote.nl [2]. The remaining citations will be uploaded into Covidence [3]. Following a pilot test of 100 citations, titles and abstracts will then be screened by two or more independent reviewers for assessment against the inclusion criteria for the review. Potentially relevant sources will be retrieved in full. The full text of selected citations will be assessed in detail against the inclusion criteria by two or more independent reviewers. Reasons for exclusion of sources of evidence at full text that do not meet the inclusion criteria will be recorded and reported in the scoping review. Any disagreements that arise between the reviewers at each stage of the selection process will be resolved through discussion, and if needed with an additional author. The results of the search and the study inclusion process will be reported in full in the final scoping review and presented in a PRISMA flow diagram [4].

# Data extraction

Data will be extracted from papers included in the scoping review by one of the review authors using a data extraction form developed by the authors. The data extracted will include specific details about the study, participants, concept, context, study methods and key findings relevant to the review questions such as the goal of the study and the implementation outcomes. A draft extraction form is provided (see Appendix B). The draft data extraction form will be modified and revised as necessary during the process of extracting data from each included study. Modifications to the data extraction form will be detailed in the scoping review. If appropriate, authors of papers will be contacted to request missing or additional data, where required.

# Data analysis and presentation

The extracted data will be assessed based on the following three predefined elements:

(1) its possibility to be incorporated into a Workers’ Health Surveillance, that is a method of screening on (early signs) of work-related CD, consultation with a health care professional and (individual) preventive advice or feedback.

(2) effectiveness of the intervention in terms of improving sustainable work participation through modifying work-environment-related or person-related factors

(3) implementation outcomes as defined by Proctor *et al*. [5]: acceptability, adoption, appropriateness, feasibility, fidelity, implementation cost, penetration and sustainability.

The extracted data concerning the intervention and its implementation will be qualitatively analyzed and presented in a diagrammatic or tabular form. A narrative summary will accompany the diagrams and tables.

# Acknowledgements

We wish to express our gratitude to Faridi Jamaludin for her help in the search and development of the search strategies.

# Funding

This review is funded by the Lexces, the Netherlands Expertise Centre for Substance-related Occupational Diseases in the scope of subsidized research by the Government of the Netherlands concerning the prevention of Occupational Diseases.

# Author contributions

JJ, JB, ST, SK, FL, HM were involved in the conception of the review

JJ, JB, ST, SK, HM were involved in the design and coordination of the review

JJ, JB, ST, SK, HM were involved in the development of the search strategy

JJ, JB, ST, SK, FL, HM will be involved in the selection for inclusion in the review

JJ, JB, ST, SK, FL, HM will be involved in data collection of the review

JJ, JB, ST, SK, FL, HM will be involved in the analysis of data and assessment of the certainty in the bod of evidence

JJ, JB, ST, SK, FL, HM will be involved in the interpretation of the data
JJ, JB, ST, SK, FL, HM will be involved in the writing of the review

JJ drafted the review protocol; JB, ST, HM, SK, FL approved the review protocol

# Conflicts of interest

The authors declare no conflict of interest.

# References

1. Peters, M., et al., *Methodology for JBI Scoping Reviews*. 2015. p. 1-24.

2. Lobbestael, G., *DedupEndNote (Version 1.0.0) [Computer software]*. 2023: Available at <https://github.com/globbestael/DedupEndNote>

3. *Covidence systematic review software*. Veritas Health Innovation, Melbourne, Australia: Available at [www.covidence.org](https://amsterdamumc.sharepoint.com/sites/NCvB/Gedeelde%20documenten/%23LEXCES%20projecten/PMO%20Huid/2-PROTOCOL/2.0%20Algemeen/2.0.1%20Origineel/2.0.1.1%20Scoping%20Review/www.covidence.org).

4. Page, M.J., et al., *The PRISMA 2020 statement: an updated guideline for reporting systematic reviews.* BMJ, 2021. **372**: p. n71.

5. Proctor, E., et al., *Outcomes for implementation research: conceptual distinctions, measurement challenges, and research agenda.* Adm Policy Ment Health, 2011. **38**(2): p. 65-76.

# Appendices

**Appendix I: Search strategy**

**21-5-2024**:

| Databases: | Before deduplication | After deduplication* |
| --- | --- | --- |
| Medline (Ovid), Embase (Ovid),  Cochrane CDSR en CENTRAL  CINAHL (Ebsco) |  |  |
| Total | 9066 | 6948 |

*Deduplication with: Dedupendnote.nl - Lobbestael, G. (2023). DedupEndNote (Version 1.0.0) [Computer software]. <https://github.com/globbestael/DedupEndNote>

MEDLINE (OVID):

Database(s): Ovid MEDLINE(R) ALL 1946 to May 20, 2024
Search Strategy:

| # | Searches | Results |
| --- | --- | --- |
| 1 | *skin diseases/ or *dermatitis/ or exp Dermatitis, Contact/ or dermatitis, atopic/ or exp foot dermatoses/ or hand dermatoses/ or leg dermatoses/ or exp skin diseases, eczematous/ or exp eczema/ | 144031 |
| 2 | ((allerg* or contact or occupation* or work*) adj6 (dermatitis or dermatos* or eczema)).ti,ab,kf. | 28675 |
| 3 | (irritant* and dermatitis).ti,ab,kf. | 3051 |
| 4 | 1 or 2 or 3 | 151151 |
| 5 | exp Occupational Exposure/ or exp Occupational Groups/ or exp Workplace/ | 825511 |
| 6 | exp Irritants/ or Industrial oils/ae or Solvents/ae or exp Hair Preparations/ae | 19271 |
| 7 | (occupation* or worker* or employee* or workplace* or work-place* or worksite or work-related or labourer* or laborer* or staff or personnel or hairdresser* or nurs* or cleaner* or painter* or surgeon* or cooks or fishing or fisher* or wet work or wet exposur* or hair dye* or hair preparation* or occupational exposure).ti,ab,kf. | 1515202 |
| 8 | (work adj3 expos*).ti,ab,kf. | 5538 |
| 9 | ((irritant* or irritative or phototoxic* or toxic* or detergent* or cutting fluid* or dyes or chemicals or cleaning agent* or chlorine or latex or disinfectant* or glove*) adj3 (expos* or occupation* or work*)).ti,ab,kf. | 28263 |
| 10 | 5 or 6 or 7 or 8 or 9 | 2059073 |
| 11 | exp Preventive Health Services/ or exp health education/ or exp primary prevention/ or secondary prevention/ or tertiary prevention/ or risk reduction behavior/ or population surveillance/ or public health surveillance/ or health plan implementation/ or national health programs/ or harm reduction/ or health behavior/ or health risk behaviors/ or risk reduction behavior/ or Occupational Health/ or *Occupational Diseases/pc or preventive medicine/ | 898229 |
| 12 | (prevent or prevention* or preventive or health program* or health care program* or programm* or health promotion or protection education or protective measur* or protective equipment* or protective glove* or skin protection* or educational intervention* or health education* or patient education* or behavio?r change* or behavio?ral change* or risk reduction* or harm reduction* or surveillance or screening).ti,ab,kf. | 2658128 |
| 13 | ((skin or dermal) adj3 intervention*).ti,ab,kf. | 942 |
| 14 | 11 or 12 or 13 | 3203312 |
| 15 | 4 and 10 and 14 | 3034 |
| 16 | (exp Animals/ or exp Animal Experimentation/ or exp models, animal/ or (rat or rats or mice or mouse or murine or murines or rodent or rodents or rabbit or rabbits or cat or cats or dog or dogs or pig or pigs or cow or cows or monkey or monkeys or goat or goats or horse or horses or ape or apes or gorilla or gorillas or sheep or sheeps or ovine or lamb or swine or swines or porcine or pup or pups or canine or beagle).ti,ab,kf.) not Humans/ | 5581502 |
| 17 | 15 not 16 | 2930 |
| 18 | exp Pediatrics/ or exp Geriatrics/ or (child* or pediatric* or paediatric* or infan* or neonate* or newborn* or baby or babies or geriatric* or older person* or older people* or older adult*).ti. | 1582907 |
| 19 | 17 not 18 | 2791 |
| 20 | (exp Child/ or exp Infant/) not Adult/ | 2012092 |
| 21 | 19 not 20 | 2729 |

EMBASE (OVID):

Database(s): Embase Classic+Embase 1947 to 2024 May 20
Search Strategy:

| # | Searches | Results |
| --- | --- | --- |
| 1 | *occupational skin disease/ or *dermatitis/ or exp contact dermatitis/ or atopic dermatitis/ or exp occupational eczema/ or hand eczema/ | 112241 |
| 2 | ((allerg* or contact or occupation* or work*) adj6 (dermatitis or dermatos* or eczema)).ti,ab,kf. | 45520 |
| 3 | (irritant* and dermatitis).ti,ab,kf. | 5199 |
| 4 | 1 or 2 or 3 | 129003 |
| 5 | exp occupational exposure/ or exp named groups by occupation/ or workplace/ | 2900412 |
| 6 | exp irritant agent/ or oil/ae or solvent/ae | 9332 |
| 7 | (occupation* or worker* or employee* or workplace* or work-place* or worksite or work-related or labourer* or laborer* or staff or personnel or hairdresser* or nurs* or cleaner* or painter* or surgeon* or cooks or fishing or fisher* or wet work or wet exposur* or hair dye* or hair preparation* or occupational exposure).ti,ab,kf. | 2048046 |
| 8 | (work adj3 expos*).ti,ab,kf. | 7384 |
| 9 | ((irritant* or irritative or phototoxic* or toxic* or detergent* or cutting fluid* or dyes or chemicals or cleaning agent* or chlorine or latex or disinfectant* or glove*) adj3 (expos* or occupation* or work*)).ti,ab,kf. | 36703 |
| 10 | 5 or 6 or 7 or 8 or 9 | 4088440 |
| 11 | preventive health service/ or prevention/ or primary prevention/ or secondary prevention/ or tertiary prevention/ or exp health education/ or education program/ or exp health program/ or exp health promotion/ or protection/ or skin protection/ or exp protective equipment/ or health behavior/ or behavior change/ or harm reduction/ or risk reduction/ or population surveillance/ or public health surveillance/ or occupational safety/ or occupational health/ or intervention study/ or prevention study/ or "prevention and control"/ or preventive medicine/ or *occupational skin disease/pc or occupational exposure/pc | 1532363 |
| 12 | (prevent or prevention* or preventive or health program* or health care program* or programm* or health promotion or protection education* or protective measur* or protective equipment* or glove* or skin protection* or educational intervention* or health education* or patient education* or behavio?r change* or behavio?ral change* or risk reduction* or harm reduction* or surveillance or screening).ti,ab,kf. | 3635717 |
| 13 | ((skin or dermal) adj3 intervention*).ti,ab,kf. | 1342 |
| 14 | 11 or 12 or 13 | 4503901 |
| 15 | 4 and 10 and 14 | 6278 |
| 16 | (exp animal/ or exp animal experiment/ or exp experimental animal/ or exp animal model/ or nonhuman/ or (rat or rats or mice or mouse or murine or murines or rodent or rodents or rabbit or rabbits or cat or cats or dog or dogs or pig or pigs or cow or cows or monkey or monkeys or goat or goats or horse or horses or ape or apes or gorilla or gorillas or sheep or sheeps or ovine or lamb or swine or swines or porcine or pup or pups or canine or beagle).ti,ab,kf.) not human/ | 8604881 |
| 17 | 15 not 16 | 6145 |
| 18 | exp pediatrics/ or exp geriatrics/ or (child* or pediatric* or paediatric* or infan* or neonate* or newborn* or baby or babies or geriatric* or older person* or older people* or older adult*).ti. | 2098685 |
| 19 | 17 not 18 | 5707 |
| 20 | (exp child/ or exp infant/) not adult/ | 2662789 |
| 21 | 19 not 20 | 5513 |

Cochrane Library:

[Cochrane Database of Systematic Reviews](https://www.cochranelibrary.com/)

Issue 5 of 12, May 2024

[Cochrane Central Register of Controlled Trials](https://www.cochranelibrary.com/)

Issue 4 of 12, April 2024

ID Search Hits

#1 ((allerg* or contact or occupation* or work*) near/6 (dermatitis or dermatos* or eczema)):ti,ab,kw 2733

#2 (irritant* and dermatitis):ti,ab,kw 548

#3 (contact dermatitis or atopic dermatitis or eczema or foot dermatoses or hand dermatoses or leg dermatoses):ti,ab,kw 10502

#4 #1 or #2 or #3 10750

#5 (occupational exposure or occupation* or worker* or employee* or workplace* or work-place* or worksite or work-related or labourer* or laborer* or staff or personnel or hairdresser* or nurs* or cleaner* or painter* or surgeon* or cooks or fishing or fisher* or wet work or wet exposur* or hair dye* or hair preparation*):ti,ab,kw 151574

#6 ((irritant* or irritative or phototoxic* or toxic* or detergent* or cutting fluid* or dyes or chemicals or cleaning agent* or chlorine or latex or disinfectant* or glove*) near/3 (expos* or occupation* or work*)):ti,ab,kw 1431

#7 #5 or #6 152640

#8 (prevent or prevention* or preventive or health program* or health care program* or programm* or health promotion or protection education* or protective measur* or protective equipment* or glove* or skin protection* or educational intervention* or health education* or patient education* or behavior change* or behavioral change* or behaviour change* or behavioural change* or risk reduction* or harm reduction* or surveillance or screening):ti,ab,kw 508660

#9 #4 and #7 and #8 in Cochrane Reviews, Trials 328

#10 (child* or pediatric* or paediatric* or infan* or neonate* or newborn* or baby or babies or geriatric* or older person* or older people* or older adult*):ti 155532

#11 #9 NOT #10 267

CINAHL (EBSCO):

557 results

( (MH "Skin Diseases") AND (MH "Occupational Diseases") ) OR ( OR (MH "Dermatitis, Contact") OR ( TI ( (allerg* or contact or occupation* or work or worker*) N3 (dermatitis or dermatos* or eczema) ) OR AB ( (allerg* or contact or occupation* or work or worker*) N3 (dermatitis or dermatos* or eczema) ) ) OR ( TI (foot dermatoses or hand dermatoses or leg dermatoses ) OR AB ( contact dermatitis foot dermatoses or hand dermatoses or leg dermatoses ) ) OR ( TI ( irritant* and dermatitis ) OR AB ( irritant* and dermatitis ) ) ) ) ) AND ( ( MH "Occupational Exposure" OR (MM "Occupational Diseases")  OR (MH "Named Groups by Occupation+") OR (MH "Solvents+/AE") OR (MH "Hair Preparations/AE") OR (MH "Cleaning Compounds/AE") OR (MH "Occupational Hazards/AE")  OR ( TI ( occupation* or worker* or employee* or workplace* or work-place* or worksite or work-related or labourer* or laborer* or staff or personnel or hairdresser* or nurs* or cleaner* or painter* or surgeon* or cooks or fishing or fisher* or wet work or wet exposur* or hair dye* or hair preparation* or occupational exposure) OR AB ( occupation* or worker* or employee* or workplace* or work-place* or worksite or work-related or labourer* or laborer* or staff or personnel or hairdresser* or nurs* or cleaner* or painter* or surgeon* or cooks or fishing or fisher* or wet work or wet exposur* or hair dye* or hair preparation* or occupational exposure) ) OR ( TI work N3 expos* OR AB work N3 expos* ) OR ( TI ( (irritant* or irritative or phototoxic* or toxic* or detergent* or cutting fluid* or dyes or chemicals or cleaning agent* or chlorine or latex or disinfectant* or glove*) N3 (expos* or occupation* or work*) ) OR AB ( (irritant* or irritative or phototoxic* or toxic* or detergent* or cutting fluid* or dyes or chemicals or cleaning agent* or chlorine or latex or disinfectant* or glove*) N3 (expos* or occupation* or work*) ) ) ) AND ( ( MH "Preventive Health Care") OR (MH "Occupational Exposure/PC") OR (MM "Occupational Diseases/PC") OR (MM "Skin Diseases/PC")  OR (MH "Health Education") OR (MH "Health Promotion") OR (MH "Preventive Trials") OR (MH "Intervention Trials") OR (MH "Harm Reduction") OR (MH "Sun Protective Behavior") OR (MH "Health Behavior") OR (MH "Occupational Health") OR ( TI ( prevent or prevention* or preventive or health program* or health care program* or programm* or health promotion or protection education* or protective measur* or protective equipment* or glove* or skin protection* or educational intervention* or health education* or patient education* or behavior change* or behavioral change* or behaviour change* or behavioural change* or risk reduction* or harm reduction* or surveillance or screening ) OR ( prevent or prevention* or preventive or health program* or health care program* or programm* or health promotion or protection education* or protective measur* or protective equipment* or glove* or skin protection* or educational intervention* or health education* or patient education* or behavior change* or behavioral change* or behaviour change* or behavioural change* or risk reduction* or harm reduction* or surveillance or screening ) ) )

NOT

(MH "Pediatrics+")  OR (MH "Geriatrics+")  OR TI (child* or pediatric* or paediatric* or infan* or neonate* or newborn* or baby or babies or geriatric* or older person* or older people* or older adult*)

**Appendix II: Data extraction instrument**

| **Category** | **Type of data** |
| --- | --- |
| 1. Bibliographic information | 1. Author(s) 2. Year of publication 3. Country of study 4. Study type |
| 1. Information concerning inclusion criteria | 1. Intervention (as named in article) 2. Target population 3. Aims of the study |
| 1. Information concerning the intervention | 1. Intervention type (primary, secondary or tertiary) 2. Intervention method(s) 3. Number of participants 4. Intervention outcomes |
| 1. Information concerning the implementation | 1. Inclusion and adherence rate/dose delivered 2. Self-reported study limitations 3. Implementation effectiveness using criteria by Proctor *et al* [5]*.* |
