# Supplementary material for: Interventions and Implementation Strategies for Preventing Occupational Contact Dermatitis: A Scoping Review
Source: Contact Dermatitis. 2026 Feb 15;94(5):437–64. doi: 10.1111/cod.70113 (PMC13070721; doi:10.1111/cod.70113)
Supplement: Supplementary file 2 — Table on study characteristics. Table S1: Characteristics of the included studies. [file COD-94-437-s002.docx]

**Article name:** Interventions and implementation strategies for preventing occupational contact dermatitis: a scoping review

**Journal name:** Contact Dermatitis

**Authors’ information:** Jonathan A.G. Jonker^12^*, Sietske J. Tamminga^12^, Felicia S. Los^12^, Parel M.V. Janse^12^, Sanja Kezic^12^ , Henk F. van der Molen^12^, Julitta S. Boschman^12^

^1^Amsterdam UMC location University of Amsterdam, Public and Occupational Health, Meibergdreef 9, Amsterdam, The Netherlands

^2^Amsterdam Public Health Research Institute, Societal Participation and Health

(*Corresponding author: j.a.g.jonker@amsterdamumc.nl)

**Supplementary file 2. Table on study characteristics.**

Table S1. Characteristics of the included studies.

| Country | Number of studies (%) | Country | Number of studies (%) |
| --- | --- | --- | --- |
| Germany | 34 (43.0) | Denmark | 13 (16.5) |
| Canada | 7 (8.9) | Italy | 5 (6.3) |
| United States | 4 (5.1) | United Kingdom | 3 (3.8) |
| Other | 13 (16.5) |  |  |
| Study design | Number of studies (%) |  | Number of studies (%) |
| Prospective cohort study | 43 (54.4) | Randomized controlled trial | 16 (20.3) |
| Pretest - Posttest | 7 (8.9) | Retrospective cohort study | 4 (5.1) |
| Non randomized comparative trial | 3 (3.8) | Other | 6 (7.6) |
| Study population | Number of studies (%) | Study population | Number of studies (%) |
| Mixed | 25 (31.6) | Healthcare workers | 24 (30.4) |
| Metal workers | 8 (10.1) | Hairdressers | 7 (8.9) |
| Food or catering workers | 7 (8.9) | Other | 8 (10.1) |
