# Supplementary material for: Interventions and Implementation Strategies for Preventing Occupational Contact Dermatitis: A Scoping Review
Source: Contact Dermatitis. 2026 Feb 15;94(5):437–64. doi: 10.1111/cod.70113 (PMC13070721; doi:10.1111/cod.70113)
Supplement: Supplementary file 3 — Overview of Data Items. [file COD-94-437-s001.docx]

**Article name:** Interventions and implementation strategies for preventing occupational contact dermatitis: a scoping review

**Journal name:** Contact Dermatitis

**Authors’ information:** Jonathan A.G. Jonker^12^*, Sietske J. Tamminga^12^, Felicia S. Los^12^, Parel M.V. Janse^12^, Sanja Kezic^12^ , Henk F. van der Molen^12^, Julitta S. Boschman^12^

^1^Amsterdam UMC location University of Amsterdam, Public and Occupational Health, Meibergdreef 9, Amsterdam, The Netherlands

^2^Amsterdam Public Health Research Institute, Societal Participation and Health

(*Corresponding author: j.a.g.jonker@amsterdamumc.nl)

**Supplementary file 3. Overview of Data Items.**

1. Author(s)
2. Year of publication
3. Country of study
4. Study type
5. Intervention (as named in article)
6. Target population
7. Aims of the study
8. Intervention type (primary, secondary or tertiary)
9. Intervention method(s)
10. Number of participants
11. Intervention outcomes
12. Inclusion and adherence rate / dose delivered
13. Self-reported study limitations
14. Implementation strategies
15. Implementation outcomes
